# Patients with carotid atherosclerosis who underwent or did not undergo carotid endarterectomy: outcome on mood, cognition and quality of life

**DOI:** 10.1186/s12888-015-0663-y

**Published:** 2015-11-12

**Authors:** Mauro Giovanni Carta, Maria Efisia Lecca, Luca Saba, Roberto Sanfilippo, Elisa Pintus, Michela Cadoni, Federica Sancassiani, Maria Francesca Moro, Davide Craboledda, Chiara Lo Giudice, Gabriele Finco, Mario Musu, Roberto Montisci

**Affiliations:** Department of Public Health and Clinical and Molecular Medicine, University of Cagliari, Cagliari, Italy; Center for Liaison Psychiatry, Psychosomatics, Azienda Ospedaliero Universitaria (A.O.U.) of Cagliari, Cagliari, Italy; Department of Radiology, Azienda Ospedaliero Universitaria (A.O.U.), of Cagliari, Polo di Monserrato s.s. 554, Monserrato, 09045 Cagliari, Italy; Department of Vascular Surgery, Azienda Ospedaliero Universitaria (A.O.U.), of Cagliari, Polo di Monserrato, s.s. 554 Monserrato, Cagliari, 09045 Italy; Department of Medical Science, University of Cagliari, Cagliari, Italy

**Keywords:** Carotid atherosclerosis, Carotid endarterectomy, Depressive disorders, Cognitive impairment, Quality of life

## Abstract

**Background:**

To compare the six-month outcome on mood, cognition and quality of life (QoL) in patients with severe carotid atherosclerosis (CA) who underwent carotid endarterectomy (CEA) with subjects who refused treatment.

**Methods:**

Cohort study on consecutive inpatients with CA (stenosis ≥ 50 %) (*N =* 46; age 72.56 ± 7.26; male 65.2 %). Intervention cohort: subjects who decided to undergo CEA (*N =* 35); Control cohort patients who refused CEA (*N =* 11). DSM-IV-Psychiatric diagnosis made by clinicians using interviews, QoL measured by Short Form Health Survey (SF-12); cognitive performance by WAIS Intelligent Coefficient (IC).

**Results:**

The study showed a better improvement during six months in Overall IC, Performance IC and Verbal IC in the group that underwent CEA. QoL in the two cohorts did not reach statistical significance. Percentages of patients who improved in the CEA group were significantly higher with regard to Overall and Verbal IC scores, and at the limits of statistical significance in Performance IC. The differences of subject with improvement in SF-12 score in the two groups did not reach statistical significance. Ages below 68 were found to be determinant of a good outcome in Overall IC score. Limit: study conducted with a small sample size.

**Conclusions:**

Patients with severe carotid atherosclerosis who underwent CEA enhanced their cognitive performance.

## Background

Atherosclerosis is a major global issue. While other causes of mortality have diminished and life expectancy has increased worldwide, the same is not true for the former [1, 2]. In fact, atherosclerotic has been the first cause of mortality in developed countries over the past decades, but it is estimated that by 2020 cardiovascular diseases and atherosclerosis will be the major causes of death also in most developing countries, including China and India [1, 2]. In this framework, carotid atherosclerosis plays an important role because is a determinant of acute cerebrovascular events such as stroke, the incidence of which in the United States is about 800,000 per year [3]. Carotid atherosclerosis is also a well-known determinant of early cognitive impairment, a factor associated with low quality of life and life expectancy in the elderly [4].

The prevalence of carotid stenosis is about 10 % in subjects over 70 years of age in the community. The majority of them present no symptoms of cardiovascular disease and report no acute vascular events [5]. Carotid stenosis can be appropriately treated and cerebral vascular attacks and stroke can be prevented. Both carotid endarterectomy (CEA), which was the gold standard treatment for many years in the past, and stenting of carotid lesions (CAS) [6, 7], a more recent alternative, are today considered to have similar efficacy and safety [8]. However, the appropriate treatments are frequently not well accepted by patients, probably owing to the fact that the disorder is asymptomatic and the patient does not always feel the need for treatment [9].

A relevant factor associated with low compliance may be comorbid depressive and mood disorders. The role of these disorders as a determinant of negative outcomes in treated patients also needs to be clarified. In fact, around 20–35 % of patients with carotid atherosclerosis have been found comorbid with major depressive or mood disorders [10–12]. Comorbidity with these disorders has been shown to be associated with poorer clinical outcomes, mortality, low functional disabilities and quality of life [12, 13]. Even though an effective treatment of depression has been found to improve the course of coronary heart disease and decrease acute events [13] with patients’ adherence to procedures, studies on the role of mood disorders concerning compliance with treatment for atherosclerosis and carotid atherosclerosis are lacking.

The co-occurrence of atherosclerosis (and neuro-vascular diseases in particular) and mood disorders may influence the evolutionary paths of both diseases [14].

The current concept of vascular depression underlines these links. Although up to now there is no agreement on diagnostic criteria for vascular depression [15, 16], they should be characterized by: 1) late-life onset of depression; 2) hyperintensities in the brain revealed by Magnetic Resonance Imaging (MRI); 3) severe cognitive impairment with deficits in executive functioning; 4) poor response to antidepressants.

The objective of this work is to perform a study of patients with severe carotid atherosclerosis (CA) eligible for carotid endarterectomy (CEA) in the six-month outcome on mood, cognition and quality of life (QoL), comparing subjects who underwent surgery with those who refused treatment.

## Methods

### Design

Cohort study

### Subjects

Subjects (Table 1) were 46 consecutive inpatients with carotid atherosclerosis at the Thoracic and Vascular Surgery Clinic of the University Hospital of Cagliari, Italy, recruited from July 2013 to May 2014 (refusal rate 22 %). The inclusion criteria were: age ≥50 years; symptomatic carotid stenosis above 50 % and/or carriers of carotid stenosis > 70 % even asymptomatic. Exclusion criteria were: contraindications to surgical procedures; cognitive deficits making the study assessment impossible.

**Table 1:** Recruited sample and Cohort study sample. Comparison by age and gender

	Recruited sample (*N =* 46)	Dropouts at t1 (*N =* 6)	Intervention cohort (*N =* 30)	Control cohort (*N =* 10)
Age^a^ (years) Mean ± ds	72.56 ± 7.26	70.36 ± 7.9	73.16 ± 7.16	72.10 ± 8.75
Gender^b^				
m	30 (65.2 %)	5 (83.3 %)	17 (56.7 %)	8(80 %)
f	16 (34.8 %)	1 (16.6 %)	13 (43,3 %)	2(20 %)

^a^Dropouts at t1 (6) vs Overall Study Sample (40) F = 0.56, DF = 1,44,45, *P =* 0.455; Intervention (30) vs Control (10) F = 0.14, *P =* 0.71,DF = 1,38,39

^b^Dropouts at t1 (6) vs Overall Study Sample (40) χ2 (with Yates corr.) = 0.29, DF = 1, *P =* 0.59; Intervention (30) vs Control (10); χ2 = 2.22, DF = 1, *P =* 0.26

Two cohorts were formed from the study sample: 1) subjects who agreed and underwent CEA were the Intervention Cohort; 2) subjects who despite the diagnosis rejected surgery formed the Control Cohort.

### Evaluation

At their entrance to the hospital ward all patients underwent a standardized diagnosis of atherosclerosis. If eligible for the study (and to undergo CEA), they were given an explanation of the purpose of the study and were required to give written consent. Those who agreed to participate were subjected to a standardized assessment of cognitive performance, quality of life and depressive symptoms at study entry (t0). Based on the fact of accepting or not accepting to undergo CEA, the sample was subdivided into the two cohorts. In the week after, those who had agreed to undergo CEA were operated on.

The same evaluation of cognitive performance, mood and quality of life was repeated six months (t1) after the first evaluation both in patients who underwent CEA and in those who had refused surgery.

The reassessment was made over two months because of difficulties in contacting patients for administration of the retest: the patients enrolled were often lost to follow-up surgery at 6 months because the University Hospital is a center of excellence and patients living far away went to local services for checkups after CEA. However, the evaluation conducted over a 2-month follow-up (6–7 months) allows a comparison in which one cannot assume serious discrepancies due to timing.

### Psychiatric assessment

The recruited subjects were interviewed by clinicians (physicians or psychologists with at least 2 years of experience in psychiatry) by means of the “Advanced Neuropsychiatric Tools and Assessment Schedule” (ANTAS) [17], a semi-structured tool derived in part from the Structured Clinical Interview (SCID) [18]. It allows the pronouncing of a psychiatric diagnosis according to DSM-IV criteria [APA 1994]. The diagnoses derived from the ANTAS were compared for reliability against those from the SCID, finding an agreement by means of Cohen’s K = 0.85 [19]. Bipolar Spectrum Disorders (BDs) were screened by means of the Mood Disorder Questionnaire (MDQ), Italian version [20]. The Hamilton Depression Rating Scale (HAM - D) [21] was used for the assessment of depressive symptoms.

### Diagnosis of carotid atherosclerosis

The diagnosis of carotid stenosis in the recruited sample was performed through clinical examination and Duplex ultrasound scanning of the epi-aortic trunks and Contrast Enhancement Computed Tomography (CECT) of the neck [22].

### Evaluation of cognitive performance

The WAIS-R (Wechsler Adult Intelligence Scale, Revised 1997) [23] consists of eleven sub-tests; six are used for the evaluation of the verbal coefficient of intelligence and form the verbal scale (Verbal IC) and five for the measurement of practical intelligence and form the performance scale (Performance IC) [23]. The sub-tests of the verbal scale are: information, memory of numbers, vocabulary, arithmetic reasoning, understanding and analogies. Those of the performance scale consist of picture arrangement, picture completion, block design, object assembly and digit symbol. Overall, the test consists of 166 questions and the overall score is used for a general measure of intelligence (Overall IC).

### Measure of quality of life

The perception of the quality of life in each subject of the sample was measured by means of the Short Form Health Survey (SF-12) [24]. The SF-12 is a widely used tool that measures the perception of quality of life according to physical activity, health problems inducing limitation of activities and goals, emotional status, pain, perception of general health, vitality, effectiveness of the social network and mental health. The score is referred for a period of one month prior to evaluation. Higher scores on the SF-12 indicated a higher perception of quality of life.

### Data analysis

Comparison between means and standard deviations of SF-12 scores and other numerical data between the two cohorts at the start of the follow-up was carried out by means of ANOVA one-way statistics and for nonparametric variables by the χ^2^ test.

In line with the stated goals, the two Main Outcome Measures of the study were: 1) comparison of the average mean in the two cohorts of the Hamilton, WAIS and SF-12 scales at the beginning and end of the trial; 2) comparison of the proportion of patients achieving a clinically relevant improvement in both groups (Improvement Rate). In keeping with the international literature, the following were considered significant improvements: 30 % decrease in the score of the Hamilton at least a 5-point increase in the SF-12; at least a 10-point increase in the total score of WAIS and WAIS verbal and performance. Regarding depressive symptomatology, the proportion of patients with a Hamilton score above or equal to 14, indicative of depressive symptoms with clinical relevance, was also compared over time in the two cohorts. The comparison of the average scores of Hamilton, WAIS, SF-12 at t0 and at the end of the trial was conducted with multivariate analysis of variance for repeated data (MANOVA). The comparison of the proportion of patients with Hamilton score ≥ 14 in the two cohorts was carried out by means of the method of Siegel and Castellan [25].

The correlation between the scores of Overall IC and SF12 was carried out by means of Pearson’s coefficient.

The Castellan test was conducted using scripts developed ad hoc with R statistical software (R Development Core Team, 2010). Other statistical analyses were processed with SPSS 13.0.1 software.

## Ethical aspects

The independent ethical committee of the Azienda Ospedaliero Universitaria of Cagliari, Italy, approved this study protocol. Each candidate signed an informed consent. The study, due to its observational design, did not imply any change in the scheduled and proposed treatment of patients. Treatment was defined according to the clinical judgment and international guidelines and by patients’ decision to accept the treatment or not.

## Results

The recruited sample (see Table 1) consisted of 46 patients with carotid stenosis; it was composed of 30 men (65.2 %) and 16 women (34.8 %).

The sample was subsequently divided into two cohorts:

The first cohort consisted of 35 patients undergoing surgery for carotid endarterectomy. This cohort consisted of symptomatic patients with stenosis above 50 % (*n =* 11) and/or asymptomatic patients with carotid stenosis > 70 % (*n =* 24).

The second cohort consisted of 11 subjects, including symptomatic patients with stenosis above 50 % (*n =* 4) and/or asymptomatic patients with carotid stenosis > 70 % (*n =* 7), who, despite the diagnosis, rejected CEA.

Of the symptomatic cases,9 (25.7 %) in the cohort undergoing surgery and 2 (18.1 %) in the control cohort showed Transient Ischemic Attack (TIA) (χ 2 with Yates correction = 0.011, *P =* 0.986, 1DF); 2 subjects (5.7 %) in the cohort undergoing surgery and 2 (18.1 %) in the control cohort showed Stroke (χ 2 with Yates correction = 0.444, *P =* 0.505, Degree of Freedom [DF] = 1).

The two cohorts did not differ in the severity of stenosis (50-70 % symptomatic or ≥70 % even asymptomatic); the two groups were homogeneous in all outcome measures found at time 0 (Hamilton, SF-12; Overall IC; Verbal IC and Performance IC scores) or in distribution by age (Table 1, age F = 0.14, *P =* 0.71,DF = 1,38,39) or gender (Table 1, χ2 = 2.22, DF = 1, *P =* 0.26).

Patients belonging to the two cohorts were contacted at a distance of 6–7 months for the evaluation (t1). Only 40 subjects (87 %) arrived for the t1 evaluation owing to six dropouts, 5 in the intervention group [14.3 % of the initial group] and 1 in the intervention rejecting group [9.1 % of this initial group]: two patients were unable to be evaluated owing to health problems that occurred after surgery not connected with vascular disease (one person was diagnosed with cancer of the cardia in September 2014 and it was impossible to fix an appointment after considering the serious health condition, the second subject was unavailable as hospitalized for abdominal surgery in the period in which the retest was to be administered); while the other four subjects refused to be re-evaluated without giving specific reasons.

The final sample (*n =* 40) of patients did not differ for age (Tab. 1, F = 0.14, DF = 1,38,39, *P =* 0.71) or gender (Tab.1, χ2 with Yates correction =2.22, DF = 1, *P =* 0.26) or for severity of the stenosis (Table 1, χ2 with Yates correction = 0.01, DF = 1, *P =* 0.99) from the initial sample.

The group of participants who entered the cohort study was divided as follows: 30 (75 %) patients who underwent CEA, 17 men (56.7 %), 13 women (43.3 %), aged 73.16 ± 7.16 (Intervention Cohort); 10 (25 %) patients who refused CEA, 8 men (80 %), two women (20 %), aged 72.10 ± 8.75 (Control Cohort) (Table 1).

Table 2 shows the scores (mean ± sd) achieved by patients of the two cohorts in the outcome measures at t0 and t1 (SF12, Hamilton, Overall IC, Verbal IC; Performance IC). The two groups were homogeneous in all outcome measures found at t0 (1-way ANOVA, DF = 1,38,39): SF12 (F = 0.01; *P =* 0.96); Hamilton (F = 0.04; *P =* 0.85); Overall IC (F = 1.02; *P =* 0.32); Verbal IC (F = 0.41; *P =* 0.52); Performance IC (F = 1.20: *P =* 00.28).

**Table 2:** SF12, Hamilton, Overall IC (WAIS), Verbal IC (WAIS); Performance IC (WAIS) scores (mean ± sd ) at t0 and t1 in the two cohorts

Tool	Score t0 Intervention Cohort (CEA)	Score t1 Intervention Cohort (CEA)	Score t0 Control Cohort	Score t1 Control Cohort
SF-12	31.70 ± 7.58	32.57 ± 7.31	29.60 ± 6.93	31.30 ± 8.62
Hamilton	6.83 ± 6.44	6.37 ± 6.59	8.20 ± 8.55	7.10 ± 5.62
Overall IC	98.63 ± 17.61	105.77 ± 19.91	92.90 ± 18.57	93.40 ± 20.47
Verbal IC	100.53 ± 17.57	108.03 ± 20.09	96.40 ± 17.61	97.50 ± 18.14
Performance IC	96.80 ± 16.30	102.10 ± 17.94	90.10 ± 18.18	89.60 ± 20.81

The two groups remained homogeneous in the two evaluations, showing a homogeneous improvement over time, relative to the mean scores of SF-12 (comparison [t0-t1] by MANOVA: Time, F = 0.941, Hyp DF = 1, DF = 38, *P =* 0.338; Groups, F = 0.487, Hyp DF = 1, DF = 38, *P =* 0.490; Time x groups, F = 0.099; Hyp DF = 1, DF = 38, *P =* 0.755) and Hamilton (comparison [t0-t1] by MANOVA: Time, F = 0.678, Hyp DF = 1, DF = 38, *P =* 0.416; Groups, F = 0.218; Hyp DF = 1, DF = 38, *P =* 0.664; Time x groups, F = 0.11, Hyp DF = 1, DF = 38, *P =* 0.741); the mean score of the Overall IC showed an improvement in time with a time-effect group, with a greater increase in the CEA group compared to the control group (comparison res [t0-t1] by MANOVA: Time, F = 8.871, Hyp DF = 1, DF = 38, *P =* 0.005; Groups: F = 1.789; Hyp DF = 1, DF = 38, *P =* 0.198; Time x groups: F = 0.111, Hyp DF = 1, DF = 38, *P =* 0.014; final score 105.77 ± 19.91 vs. 93.40 ± 20:47); a similar result was evident in relation to the Verbal IC score (comparison [t0-t1] by MANOVA: Time, F = 10.569, Hyp DF = 1, DF = 38, *P =* 0.002; Groups, F = 1,206; Hyp DF = 1, DF = 38, *P =* 0.279; Time x groups, F = 5.853, Hyp DF = 1, DF = 38, *P =* 0.020) and to the Performance IC (comparison [t0-t1] by MANOVA: Time, F = 3.328, Hyp DF = 1, DF = 38, *P =* 0.076; Groups, F = 2.291, Hyp DF = 1, DF = 38, *P =* 0.138; Time x groups, F = 4.860, Hyp DF = 1, DF = 38, *P =* 0.034) (Table 2).

In the sub-cohort of those who underwent surgery, 12 people (40 %) showed an improvement in the SF-12 indicative score against 2 (20 %) in the control group (χ 2 = 1.319; df 1; *P =* 0.251). Regarding the Hamilton score in the group receiving CEA, 13 people (44.3 %) resulted improved, quantified as a decrease of 30 % in the score against two (20 %) in the control group (χ 2 = 1.742, *P =* 0.187, DF = 1).

As regards the Overall IC score in the CEA group, 10 patients (33.3 %) showed an increase of at least 10 points in the score against none in the control group (0 %) (χ 2 = 4.44, DF = 1, *P =* 0.035). As regards the Verbal IC score, 9 patients in the CEA group improved at least by 10 points (30 %), vs 0 (0 %) in the control group (χ 2 = 4.44, DF = 1, *P =* 0.049). In the improvement of Performance IC the same trend was noted, with 8 patients in group CEA who improved by at least 10 points (26.6 %%) against 0 (0 %) in the control group, but, in this case, the difference did not reach statistical significance (χ 2 = 4.44, DF = 1, *P =* 0.068).

Those with depressive symptoms (Hamilton ≥ 14) in the CEA group were 5 (16.6 %) at t0 and 4 (20 %) at t1; in the control group they were 2 (20 %) at t0 and 2 (20 %) t1. The analysis of variance for Castellan’s nominal data did not detect statistically significant differences (χ 2 = 0.8, DF = 4, *P =* 0.723).

Table 3 shows what factors (measured at t0) are associated with a positive outcome (improvement in the Overall IC test by at least 10 points). The sole determinant of outcome appears to have been the younger age. Patients who acquired a significant improvement in cognitive performance after six months from CEA were younger (on average by about 7 years) with a statistically significant difference compared to the age of those who did not achieve a good outcome in terms of cognitive performance. The presence of depressive symptoms at baseline was higher in the group with negative results, but the difference between the two groups did not reach the limits of statistical significance. The other factors (sex, presence of at least one psychiatric diagnosis DSM-IV, MDQ score, SF-12 score) were homogeneous between the two groups divided on the basis of outcome.

**Table 3:** Determinants of cognitive outcome (Positive outcome at least a 10-point increase in the total score of WAIS Overall IC)

	Gender [F] (%)	Age	SF-12	MDQ^ab^	MDQ + (%)^c^	Psychiatric Diagnosis (%)^a^	Hamilton	Hamilton ≥14 (%)
Overall IC ≥ 10	4 (40)	68.9 ± 6.2	31.8 ± 7.4	3.5 ± 2.8	1 (10)	2 (20)	6.0 ± 5.0	0 (0)
Overall IC <10	9 (45)	75.4 ± 7.7	31.7 ± 7.8	2.5 ± 4.2	3 (15)	5 (25)	7.3 ± 7.1	5 (25)
Total	13 (43)	73.2 ± 7.2	31.8 ± 7.6	2.8 ± 3.7	4 (13.3)	7 (23.3)	6.8 ± 6.4	5 (16.7)
F, ANOVA 1way (Bonferroni) DF = 1,28,29		F = 5.25^*^	F = 0.01 *P =* 0.97	F = 0.46 *P =* 0.53			F = 0.27 *P =* 0.61	
χ 2 with Yates correction DF = 1	χ2 = 0.07 *P =* 0.79				χ2 = 0.14 *P =* 0.70	χ2 = 0.09 *P =* 0.76		χ2 = 1.96 *P =* 0.16

^*^ *p <* 0.030

^a^ DSM-IV Anxiety; Mood or Eating Disorders

^b^ Mood Disorder Questionnaire mean score

^c^ Mood Disorder Questionnaire positives (score >6) in the two groups

Patients who underwent surgery with an age above 68 were 23, their mean increase in Overall IC score during the observation period was 5.65 ± 5.70; those aged 68 or less were 7, and showed an increase (t0-t1) of 12.42 ± 11.1 in the Overall IC score; the difference was statistically significant (F = 4.73; DF = 1,18,29; *P =* 0.038).

In the intervention group (*n =* 30) a correlation of the scores of Overall IC and SF12 calculated at t0 was found (Pearson’s coefficient, 58 DF, r = 0:43 *p <*0.5), while at t1 (*n =* 30), the correlation did not reach statistical significance (r = 0.145, *p >* 0.5). In the group of patients not operated this correlation did not reach statistical significance in either of the two measurements t0-t1 (Pearson’s coefficient, DF = 18, r = 0.172, *p >* 0.5; r = 0.237, *p >* 0.5).

## Discussion

This is the first study in literature that has measured the effectiveness of CEA against a control group that did not undergo the treatment. However, this was not a randomized controlled trial, but a cohort study where belonging to the control group was decided by the same patient who refused treatment.

The study showed an improvement during the study (six months) in the mean scores of Overall IC, Performance IC and Verbal IC in the whole sample of patients with carotid atherosclerosis, but the improvement was more marked in the group that underwent CEA. Perception of quality of life (SF-12 score) and depressive symptoms (Hamilton score) improved in the sample over time but the difference between the two cohorts did not reach statistical significance.

The figures are perfectly consistent with the fact that the percentages of patients who improved significantly in the group that underwent CEA were significantly higher with regard to Overall and Verbal IC score, and at the limits of statistical significance in Performance IC (subjects who improved were those with at least 10 points higher at t1 in Overall, Verbal or Performance IC).

The differences in the percentages of subjects with improvement in SF-12 (at least a 5-point increase at t1) and Hamilton score (30 % decrease) in the two groups did not reach statistical significance.

The choice to repeat the psychiatric and neuro-cognitive evaluation after 6 months was determined by the literature [19, 26], which indicates that an assessment of cognitive performance made before this time period may produce erroneous and unreliable results. This phenomenon may be a consequence of a sudden cerebral hyperperfusion in months following CEA ([26, 27].

Improvement in cognitive performance after CEA had been so far described only on the basis of anecdotal or case studies, rather than through studies with control groups. Furthermore, the results of these studies were not completely concordant [28]. An uncontrolled study showed an improvement in pre-post score WAIS, but also deterioration in the working memory [29]. In fact, in a recent systematic review it was impossible to perform the meta-analysis owing to the inhomogeneity of the measuring instruments used and the lack of studies with control groups [30].

The study by Wassek et al. [31] found a significant cognitive decline in patients ≥ 68 years, both in patients treated with CEA and in those treated with the stenting technique (CAS), but this cognitive impairment persisted in patients after CEA, while it was transient in patients treated with CAS. The data from our study are only partially consistent with this result. In our sample, patients aged under and over 68 years did in fact show an increase in the score of IC Overall, although the improvement was more pronounced among younger people (7 point score mean). It is still to be emphasized that the evaluation in Wassek’s study was conducted at 3 months, a period much shorter than ours.

The quality of life does not improve differently in the two cohorts, although the much higher improvement in the CEA group produces a tendency to a statistically significant difference between the two groups over time. It can be assumed that the improvement of quality of life needs a longer time than does improvement in cognitive performance, because the latter may be a determinant in improvement of the perception of the quality of life. In fact, on evaluating the correlation between Overall IC and SF12 scores, a positive correlation at t0 in the cohort of the intervention was shown; this correlation was not apparent at t1 in the same group of subjects who underwent surgery.

Depressive symptoms did not improve in the treated group compared to the control group, but we must emphasize that the average Hamilton scores were moderately low in the two groups and the percentage of people with a number of depressive symptoms of clinical relevance was relatively low (≥14 Hamilton score, around 20 % of the overall sample). In fact, patients with carotid atherosclerosis comorbid with major depression in a range between 20–35 % in literature [10–12, 32], thus the results of this study were at the lower limit of the range. Owing to the low power of the study, it was therefore unlikely to find differences even when they were supposedly present.

Having depressive symptomatology (Hamilton ≥14) at t0 was associated with a lower outcome (lower IC Overall score at t1), but this trend did not reach statistical significance. Although due to the limit of a small sample for refusing the null hypothesis, we can say that unlike previous studies, the results of our study can be compared through meta-analysis with those of future studies because they were collected with a standardized methodology for measuring outcomes and with a controlled design.

## Limitations

The study was conducted on a small sample, and this limits its power.

## Conclusions

This is the first study in literature that has measured the effectiveness of CEA against a control group without treatment and shows that patients with severe carotid atherosclerosis enhance their cognitive performance if they undergo CEA in comparison with patients with the same disorder who refuse surgery.

The study suggests that cognitive decline due to carotid stenosis can be appropriately prevented if the treatment for stenosis is conducted early. These results should be better explained to patients because treatments are frequently not well accepted, probably owing to the fact that the disorder is asymptomatic in this early course and the patient does not always feel the need for treatment.

Given the difficulty of conducting this type of research, it is essential for future research, even when conducted on small samples, to maintain a controlled design and standardized methods of outcome evaluation. This will allow performance of meta-analyses up to now impossible to carry out.

## Abbreviations

CEA: Carotid endarterectomy
CAS: Stenting of carotid lesions
QoL: Quality of life
ANTAS: Advanced Neuropsychiatric Tools and Assessment Schedule
SCID: Structured Clinical Interview
BDs: Bipolar Spectrum Disorders
MDQ: Mood Disorder Questionnaire
HAM – D: Hamilton Depression Rating Scale
CECT: Contrast Enhancement Computed Tomography
WAIS-R: Wechsler Adult Intelligence Scale, Revised
Verbal IC: Coefficient of verbal intelligence
Performance IC: Coefficient of practical intelligence
Overall IC: Coefficient of general intelligence
SF-12: Short Form Health Survey
ANOVA: Analysis of Variance
MANOVA: Multivariate analysis of variance for repeated data
SPSS: Statistical Package for the Social Sciences
TIA: Transient Ischemic Attack
DF: Degrees of freedom
